# Effectiveness of laser photobiomodulation in the management of trigeminal neuralgia: a systematic review and meta-analysis

**DOI:** 10.1007/s10103-026-04804-9

**Published:** 2026-02-03

**Authors:** Paula da Costa Taddeucci, Gabriel Campos Louzeiro, Karen Cherubini, Juliana Cassol Spanemberg, Fernanda Gonçalves Salum

**Affiliations:** 1 Oral Medicine Division, Pontifical Catholic University of Rio Grande do Sul, Porto Alegre, Brazil; 2 Faculty of Health Sciences, Fernando Pessoa Canarias University, Las Palmas, Spain

**Keywords:** Facial neuralgia, Facial pain, Trigeminal neuralgia, Postherpetic neuralgia, Low level laser therapy, Photobiomodulation

## Abstract

This systematic literature review aimed to assess the effectiveness of laser photobiomodulation therapy (PBM) in reducing pain in patients with trigeminal neuralgia and postherpetic neuralgia. Randomized controlled clinical trials were identified through searches in the following databases: PubMed, Web of Science, LILACS, EMBASE, Scopus, and the Cochrane Library. No studies involving patients with postherpetic neuralgia met the inclusion criteria and were therefore excluded. Nine studies, comprising a total of 387 patients diagnosed with trigeminal neuralgia, were included. In seven of these studies, patients also received pharmacological treatment, which was initiated either prior to or concurrently with the PBM intervention. A sham laser was used as a control in six studies. PBM parameters varied widely and were often incompletely reported, limiting the ability to compare treatment protocols. Qualitative analysis showed that eight out of nine studies reported a significantly greater reduction in pain scores in the PBM groups compared to control groups. A low to moderate risk of bias was found in two-thirds of the studies. The meta-analysis, which included five studies, demonstrated a significant effect of PBM in reducing pain scores (MD − 2.17; 95% CI, − 3.30 to − 1.04; *P* = 0.0002), although high heterogeneity was observed (I² = 92%). PBM appears to be an effective adjuvant therapy for trigeminal neuralgia, with a favorable safety profile and potential to reduce medication dependency. Nevertheless, the results should be interpreted cautiously due to substantial heterogeneity, lack of PBM protocol standardization, and limited long-term outcome data.

**Supplementary Information:**

The online version contains supplementary material available at 10.1007/s10103-026-04804-9.

## Introduction

Pain is widely recognized as a multidimensional phenomenon, influenced by biological, psychological, and social factors [1–3]. When pain persists for a period exceeding three months, it is defined as chronic and may arise from neuropathic, musculoskeletal, or inflammatory etiologies [4]. Neuropathic pain results from a lesion or disease affecting the peripheral or central somatosensory system. It is characterized by hyperalgesia and allodynia, in addition to a limited response to non-steroidal anti-inflammatory drugs (NSAIDs) [5–7]. Its global prevalence is estimated to range between 7% and 10% [1]. The diagnostic criteria have been established by the International Association for the Study of Pain (IASP) and the International Classification of Headache Disorders [8–10].

Trigeminal neuralgia is a neurological disorder characterized by recurrent episodes of severe, unilateral facial pain, typically described as lancinating, sharp, and paroxysmal in nature, involving one or more branches of the trigeminal nerve. The pain is often triggered by innocuous stimuli such as speaking, chewing, or light tactile contact with the facial skin, and it exerts a profound negative impact on quality of life. Etiologically, the condition may be classified as classical, when attributed to vascular compression of the trigeminal nerve; idiopathic, when no identifiable cause is found; or secondary, when associated with underlying conditions such as tumors in the cerebellopontine angle, arteriovenous malformations, or multiple sclerosis [11]. The diagnosis is predominantly clinical and based on the recognition of hallmark features, including brief paroxysms of intense pain within the trigeminal nerve distribution, typically occurring in the absence of neurological deficits. Magnetic resonance imaging (MRI) serves as an important adjunct in excluding secondary structural pathologies, such as tumors or vascular malformations. In cases where pharmacological management proves ineffective or poorly tolerated, neurosurgical interventions, most notably microvascular decompression, may be indicated as a therapeutic option [12].

Postherpetic neuralgia is the most common complication of herpes zoster and is defined by the persistence of pain for more than 90 days following the resolution of cutaneous lesions. In the facial region, it frequently involves the ophthalmic division of the trigeminal nerve, resulting in severe pain, burning sensations, hyperalgesia, and allodynia [13]. This condition predominantly affects elderly individuals and immunocompromised patients, potentially leading to significant functional and psychological impairment. The diagnosis is clinical and is established based on a prior history of herpetic infection and the continued presence of localized pain [14].

The treatment of neuropathic pain is multifaceted and often requires the use of centrally acting pharmacological agents, as these conditions respond poorly to conventional analgesics. Recommended medications include tricyclic antidepressants, anticonvulsants, and, specifically for trigeminal neuralgia, carbamazepine, despite its potential adverse effects [14, 15]. Other therapeutic alternatives encompass oxcarbazepine, baclofen, and gabapentin. A multimodal approach is also essential, integrating pharmacotherapy, physical therapy, psychotherapy, and, more recently, laser photobiomodulation therapy (PBM). Combined therapeutic strategies have been shown to yield more favorable outcomes compared to monotherapeutic interventions [16].

PBM has emerged as a promising adjuvant therapy in the management of chronic pain. Its mechanism of action involves the application of light at specific wavelengths that stimulate mitochondrial activity and enhance adenosine triphosphate (ATP) production. Additionally, PBM promotes repolarization of cell membranes and reduces the release of pro-inflammatory mediators [17]. These effects contribute to tissue regeneration, attenuation of inflammation, and the disruption of the pain–muscle spasm cycle. Clinical studies indicate that when PBM is combined with conventional treatments, such as pharmacotherapy or physical therapy, it enhances analgesic efficacy and accelerates functional recovery[18]. The technique is considered safe, non-invasive, and well tolerated, with particular relevance in dental settings due to its ease of integration into clinical practice. However, maximizing its therapeutic efficacy requires the careful selection of key parameters such as output power, wavelength, irradiance, energy density, and application frequency [17].

This systematic literature review and meta-analysis aimed to investigate the effectiveness of laser PBM in reducing pain in adult patients with trigeminal neuralgia and postherpetic neuralgia in the orofacial region.

## Materials and methods

This review was conducted in accordance with the guidelines set forth by the Preferred Reporting Items for Systematic Reviews and Meta-Analyses (PRISMA) Statement. The review protocol was registered in the International Prospective Register of Systematic Reviews (PROSPERO) under registration number CRD420251102473.

A systematic literature review of randomized controlled clinical trials was conducted to address the following research question: *Is laser PBM*,* whether used alone or in combination with other therapies*,* effective in reducing pain in patients with trigeminal neuralgia and postherpetic neuralgia in the orofacial region?*

The following information outlines the PICO question:

### Population

Adult individuals of both sexes presenting with trigeminal neuralgia or postherpetic neuralgia in the orofacial region.

### Intervention

PBM, administered either as a monotherapy or in conjunction with other treatments.

### Comparison

Placebo, sham laser, no treatment, centrally acting pharmacological agents, or any other type of intervention.

### Outcome

Reduction in pain symptoms.

### Selection of studies

Searches were conducted across the following databases: PubMed, Web of Science, LILACS, EMBASE, Scopus, and the Cochrane Library. The search strategy, which incorporated the following MeSH terms (Medical Subject Headings from MEDLINE) “Facial Neuralgia” OR “Facial Pain” OR “Trigeminal Neuralgia” OR “Postherpetic Neuralgia” AND “Laser Therapy” OR “Low Level Light Therapy,” was initially developed using the Medline/PubMed platform and subsequently adapted for the remaining databases.

Manual searches were also conducted among the most recent references on the topic, as well as within the grey literature. No filters were applied regarding language or publication date. The Rayyan software was used to facilitate the independent selection of articles by two separate reviewers (PCT, JCS). After a thorough screening of the titles and abstracts of the retrieved studies, publications that were not relevant to the research question were excluded. Studies considered potentially eligible were read in full and assessed by both reviewers according to the predefined inclusion and exclusion criteria.

## Inclusion and exclusion criteria

Aiming to increase methodological rigor, internal validity, and the reliability of the estimates, only randomized controlled clinical trials were selected. The manuscripts were selected based on the following eligibility criteria:

- adult patients with trigeminal neuralgia or postherpetic neuralgia in the orofacial region, diagnosed using validated methods such as the International Classification of Headache Disorders;
- inclusion of at least one intervention group receiving PBM, either as a standalone therapy or in combination with other treatments;
- description of the PBM protocols employed;
- assessment of pain outcomes using validated measurement scales.

Studies were excluded according to the following criteria:

- failure to assess pain levels both before and after the intervention;
- use of laser acupuncture as the intervention modality;
- patients diagnosed with secondary trigeminal neuralgia — including those with multiple sclerosis, tumors in the cerebellopontine angle, or arteriovenous malformations. Additionally, observational studies, retrospective analyses, case series, case reports, and systematic literature reviews were excluded from this review.

## Outcome assessment

The outcome of this review was the reduction in pain following treatment, as measured by validated pain assessment scales, including the Visual Analog Scale (VAS), the Numeric Rating Scale (NRS), the Brief Pain Inventory – Facial (BPI-Facial), and/or the McGill Pain Questionnaire.

## Data extraction

From the selected articles, the following data were independently extracted by two researchers: name of the first author, country of origin, year of publication, number of participants enrolled, participants’ sex and age, type of orofacial pain, and diagnostic methods employed.

Regarding laser PBM therapy, the following data were extracted: type of laser and all PBM parameters reported in the study, as well as the number and frequency of sessions, duration of treatment, and follow-up period. For pharmacological therapies, information was collected on the medications administered, including dosage, therapeutic regimen, treatment duration, and follow-up time. With respect to other non-pharmacological interventions, data were extracted on the type of intervention, therapeutic protocol, treatment duration, and follow-up period, along with any additional relevant information provided in the study.

Regarding outcomes, pain scores at baseline and after treatment were extracted. When pain assessments were conducted during short and long-term follow-up periods, these data were also collected.

## Quality rating assessment

Risk of bias was independently assessed by two researchers using the RoB 2.0 tool (Revised Cochrane risk-of-bias tool for randomized trials), the instrument currently recommended by the Cochrane Collaboration. For each outcome of interest, five domains related to potential sources of bias were evaluated: bias arising from the randomization process, deviations from the intended interventions, bias due to missing outcome data, bias in the measurement of outcomes, and bias in the selection of the reported results.

### Data analysis

The results were presented descriptively, and the evidence was summarized in tabular format. The association between PBM and pain-related outcomes was evaluated through meta-analysis. The relative effect was estimated using the mean difference (MD), calculated via the inverse variance method with 95% confidence intervals. For this analysis, the mean values of the specified outcomes and their respective standard deviations were extracted from the included studies.

To assess heterogeneity, the Chi-square (χ²) test and I-square (I²) statistic were employed, with I² values greater than 50% indicating substantial heterogeneity. In such cases, a random-effect model was applied. The high degree of heterogeneity observed among the studies was further explored through subgroup meta-analyses.

## Results

**Fig. 1:**
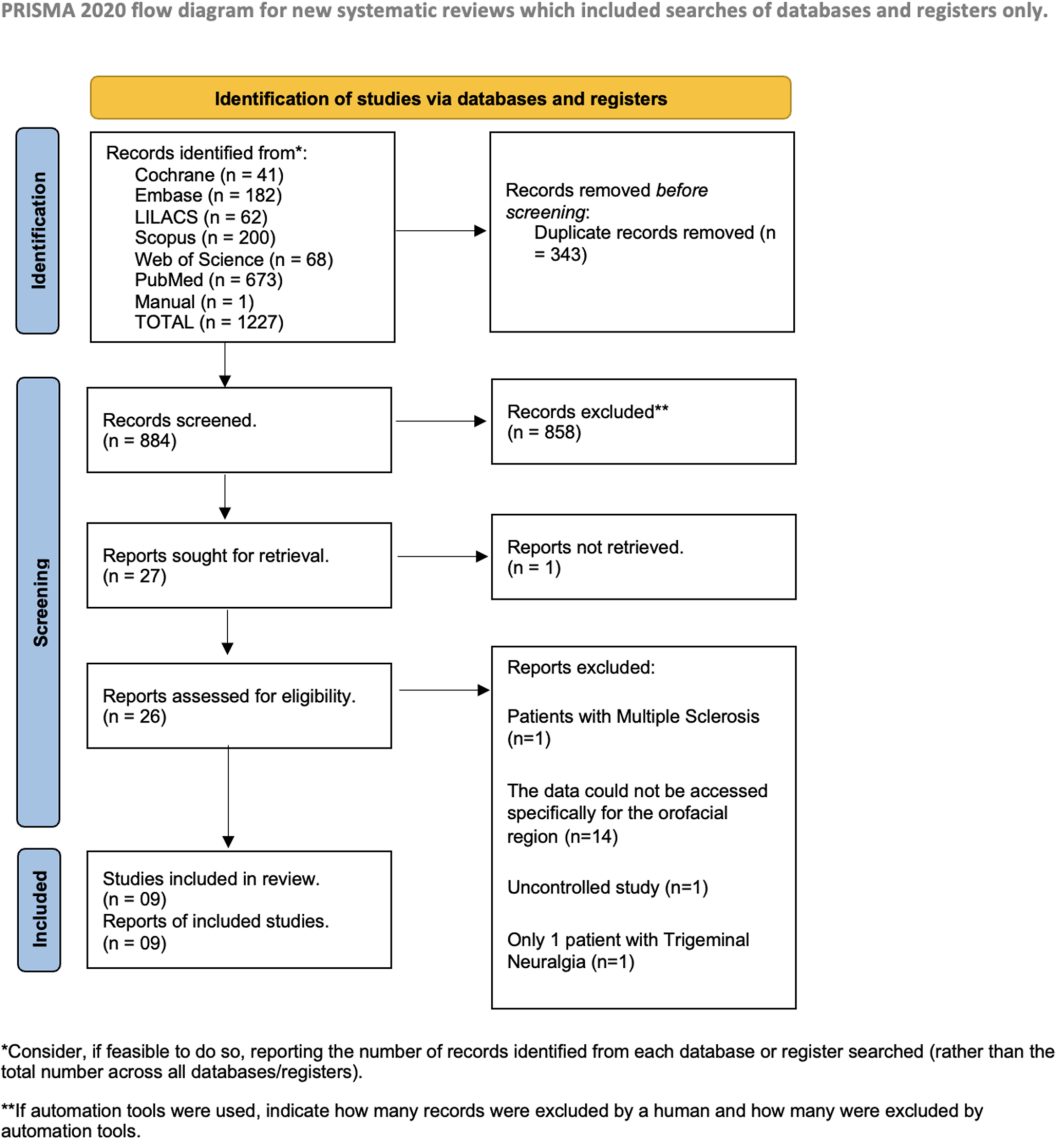
Flow Diagram of Study Selection

The final database search was conducted on July 22, 2025. A total of 1,227 articles were initially identified across the selected databases. Of these, 343 were excluded due to duplication, resulting in 884 records available for title and abstract screening. Following the application of the predefined eligibility criteria, 858 articles were excluded. Subsequently, 27 studies were selected for full-text review, and nine of these met the PICO-based inclusion criteria and were ultimately included in this systematic review. The flowchart illustrating the search and selection process is presented in Fig. 1.

Nine randomized controlled clinical trials investigating the efficacy of laser PBM in the management of trigeminal neuralgia were included in this review. No eligible studies specifically addressing postherpetic neuralgia in the orofacial region were identified. The total number of participants with trigeminal neuralgia across the included studies was 387. General characteristics of the studies are summarized in Table 1, with sample sizes ranging from 20 to 120 participants and age distributions between 18 and 72 years.

**Table 1:** General data regarding the randomized controlled trials conducted in patients with trigeminal neuralgia included in this systematic review

Author/Year/Country	Sample	Sex/Age	Study Design	Study Objectives/ Outcome Measures
Walker et al. 1987 (USA)[26]	35	**Sex**: NI **Age Range**: 18–45	Randomized Controlled Trial	To evaluate the efficacy of PBM laser in reducing pain intensity and the frequency of pain episodes. Assessment scale used: VAS.
Aghamohammadi et al. 2012 (Iran) [27]	42	**Sex**: NI **Age Range**: 30–70	Randomized Controlled Trial	To evaluate the efficacy of PBM laser in combination with trigeminal ganglion block for pain relief. Assessment scale used: VAS.
Amanat et al. 2013 (Iran)[19]	26	**Sex**: 9 M, 17 F **Mean Age**: 47.22	Randomized Controlled Trial	To evaluate the efficacy of PBM laser in pain management. Assessment scale used: VAS.
Waked et al. 2015 (Egypt) [21]	45	**Sex**: 19 M, 26 F **Age Range**: 25–50	Randomized Controlled Trial	To evaluate the efficacy of PBM laser in pain management and to compare outcomes across different laser application sites. Assessment scale used: Numerical Rating Scale.
Antonić et al. 2017 (Croatia) [20]	20	**Sex**: 12 M, 8 F **Age Range**: 27–72	Randomized Controlled Trial	To evaluate the efficacy of different wavelengths of PBM laser in pain management. Assessment scale used: VAS.
Ebrahimi et al. 2018 (Iran)[22]	30	**Sex**: 11 M, 19 F **Age**: NI	Randomized Controlled Trial	To evaluate the efficacy of PBM laser combined with carbamazepine in reducing pain symptoms. Assessment scale used: VAS.
Al-Azab et al. 2023 (Egypt) [25]	120	**Sex**: NI **Age Range**: 26–41	Randomized Controlled Trial	To compare the effects of PBM laser with those of electromagnetic therapy. Primary outcome: Amplitude of masseter and temporalis muscle action potentials. Secondary outcome: Pain intensity. Assessment scale used: VAS.
Rezazadeh et al. 2023 (Iran) [23]	24	**Sex**: NI **Age**: NI	Randomized Controlled Trial	To evaluate the efficacy of non-ablative, non-thermal CO₂ laser (NANTCL) in pain reduction. Assessment scale used: VAS.
Karagözoğlu et al. 2024 (Turkey) [24]	45	**Sex**:19 M, 26 F **Mean Age**: 46.09	Randomized Controlled Trial	To compare the efficacy of two PBM laser protocols combined with pharmacological treatment in terms of pain frequency and intensity, interference with general activities, and interference with specific facial functions. Assessment scale used: BPI-Facial.

*PBM* photobiomodulation, *M* male, *F,* female, *NANTCL* non-ablative, non-thermal CO₂ laser, *VAS* Visual Analog Scale, *BPI*-*Facial* Brief Pain Inventory – Facial, *NI* not informed.

Although the studies by Amanat et al. [19] and Antonić et al. [20] assessed different types of chronic orofacial pain, they included 26 and 20 individuals with trigeminal neuralgia, respectively. In seven studies, the diagnosis of trigeminal neuralgia was established according to the criteria of the International Headache Society [19–25]. In one study, neurologists diagnosed the condition based on physical examination findings [26]. The study by Aghamohammadi et al. [27] did not specify the diagnostic method; however, it included only patients with refractory pain.

Pain was assessed using the VAS or the NRS in all studies, except for the trial conducted by Karagözoğlu et al. [24], which employed the Brief Pain Inventory – Facial (BPI-Facial). This instrument, comprising three sections, evaluates pain intensity, interference with general activities, and impairment of orofacial functions. Pain assessment scales were administered at baseline, post-treatment, and during follow-up periods.

In two studies [20, 26], PBM was used as a standalone therapy, as participants were not receiving any pharmacological or other concomitant treatments. In seven studies, laser PBM was associated to pharmacological treatment, initiated either before or during the course of the study. Carbamazepine, administered in doses ranging from 100 to 1200 mg per day, was the drug of choice [19, 21–25, 27]. Rezazadeh et al. [23] administered carbamazepine, gabapentin, or baclofen, while Al-Azab et al. [25] used carbamazepine in combination with nefopam hydrochloride and a vitamin complex. In the study conducted by Aghamohammadi et al. [27], PBM was administered in combination with carbamazepine and trigeminal ganglion block (Table 2).

**Table 2.** Data related to methodology, laser PBM parameters, comparator treatments, and outcomes of the randomized controlled trials conducted in patients with trigeminal neuralgia

Author/Year/Country	Groups	Parameters of PBM Laser/Comparator Therapy	Study Duration /Follow Up	Outcomes
Walker et al. 1987 (USA) [26]	PBM laser (*n* = 17) Sham laser (*n* = 18)	He: Ne laser, spot size 4 mm², 1 mW output power, 632.5 nm wavelength, 47.6 mW/cm² irradiance, 30 s of exposure in the 1 st week, 45 s in the 2nd week, 60 s from the 3rd to the 6th week, and 90 s from the 7th to the 10th week, 3 sessions per week.	10 weeks (30 sessions) Follow-up: NI	Participants in the PBM laser group exhibited a significantly greater reduction in both pain intensity and the frequency of painful episodes (*P* < 0.002).
Aghamohammadi et al. 2012 (Iran) [27]	PBM laser + ganglion block + pharmacological therapy (*n* = 21) Ganglion block + pharmacological therapy (*n* = 21)	**PBM laser** 890 nm, 3 to 10 J per point applied to the mandibular notch, 12 sessions. Other PBM parameters not reported. **Ganglion block** Performed via the foramen ovale with the injection of 3 mL of 0.5% bupivacaine and 40 mg of methylprednisolone. **Pharmacological Therapy** Carbamazepine, 200 mg per day.	Up to 12 days (12 sessions) Follow-up: 6 months.	Pain intensity was significantly lower in the PBM group from the 7th day onward and throughout the entire follow-up period. The number of carbamazepine tablets taken was also significantly lower in the PBM group. Additionally, the pain-free period was significantly longer (*P* < 0.001).
Amanat et al. 2013 (Iran) [19]	PBM laser + pharmacological therapy (*n* = 13) Sham laser + pharmacological therapy (*n* = 13)	**PBM laser** Diode laser (GaAs), spot size 0.2826 cm², 0.012 W average power, 0.042 W/cm² irradiance, 980 nm wavelength, 3000 Hz, pulse duration 400 ns, 12.73 J/cm² density of energy, 3.6 J per point, 300 s of exposure at each trigger point, 3 sessions per week. Pharmacological Therapy* Carbamazepine	3 weeks (10 sessions) Follow-up: de 2 and 4 months	Both groups showed a significant reduction in pain scores at the end of treatment (*P* < 0.0001), with no significant difference between them (*P* = 0.726). Pain levels increased significantly during follow-up in both groups (*P* < 0.001).
Waked et al. 2015 (Egypt)[21]	PBM laser on trigger points + pharmacological therapy (*n* = 15) PBM laser along the nerve path + pharmacological therapy (*n* = 15) Sham laser + pharmacological therapy (*n* = 15)	**PBM laser applied to trigger points** He: Ne laser, 100 Hz, for 15 min, 3 sessions per week Other PBM parameters not reported. **PBM laser along the nerve path** He: Ne laser, 100 Hz, for 15 min, 3 sessions per week. Pharmacological Therapy* Carbamazepine	8 weeks (24 sessions) Follow-up: NI	PBM was more effective than placebo in trigeminal neuralgia and the trigger points application was superior to nerve path application (*P* < 0.05).
Antonić et al. 2017 (Croatia) [20]	PBM 660 nm (*n* = 10) PBM 810 nm (*n* = 10)	Diode laser (GaAlAs), spot size 0.2 cm, 100 mW output power, wavelengths of 660 nm and 810 nm, 3 J/cm² density of energy, scanning application for 10 min, 5 sessions per week.	4 weeks (20 sessions) Follow-up: NI	VAS scores were significantly lower after PBM in all individuals and for both wavelengths. The efficacy of the 810 nm laser was significantly greater than that of the 660 nm laser (*P* = 0.024).
Ebrahimi et al. 2018 (Iran) [22]	PBM laser + pharmacological therapy (*n* = 15) Sham laser + pharmacological therapy (*n* = 15)	**PBM laser** Diode laser (GaAlAs), spot size 1 cm in diameter, 0.2 W output power, 810 nm wavelength, 6.36 J/cm² density of energy, 5 J per point, 25 s of exposure per point. Irradiation was applied to trigger points, 3 sessions per week. **Pharmacological Therapy** 100 mg of carbamazepine at the beginning of treatment, followed by an additional 100 mg every two days until pain relief was achieved.	3 weeks (9 sessions) Follow-up: 1 month	Pain intensity decreased in both groups by the end of treatment. VAS scores were significantly lower in the PBM group (*P* = 0.003). One month after the completion of treatment, partial recurrence of symptoms was observed in both groups (*P* = 0.003).
Al-Azab et al. 2023 (Egypt) [25]	Pharmacological therapy (*n* = 40) PBM laser + pharmacological therapy (*n* = 40) Electromagnetic therapy + pharmacological therapy (*n* = 40)	**PBM laser** He: Ne laser, 15 mW output power, irradiance 150–170 mW/cm², 830 nm wavelength, 20 min of exposure at trigger points, 3 sessions per week. **Pharmacological Therapy** Glycemic control medications: gliclazide, glibenclamide, or metformin. Carbamazepine: 600–1200 mg/day Analgesic: Nefopam hydrochloride 30 mg for acute pain Vitamin complex: Benfotiamine 40 mg + Vitamin B6 60 mg + Vitamin B12 250 mcg **Electromagnetic Therapy** Electromagnetic electrodes, with each session lasting 20 min. A continuous magnetic field was applied, with a magnetic induction of 30 Gauss and a frequency of 50 Hz.	2 months (24 sessions) Follow-up: NI	A statistically significant difference was observed between the groups, with superior outcomes in the PBM group, both in pain reduction and in the improvement of CMAP amplitude (*P* = 0.001).
Rezazadeh et al. 2023 (Iran) [23]	PBM laser + pharmacological therapy (*n* = 12) Sham laser + pharmacological therapy (*n* = 12)	**PBM laser** Defocused CO₂ laser, 1.1 W output power, 10,600 nm wavelength, 100 Hz, exposure of 10 s per trigger point in the first week and 20 s in the second week, 3 sessions per week. Pharmacological Therapy* Carbamazepine, gabapentin, or baclofen.	2 weeks (6 sessions) Follow-up: 1 week, 1 month e 3 months	Both groups showed a significant reduction in pain scores (*P* < 0.05). However, the difference between the groups was statistically significant only up to one week after treatment (*P* = 0.04).
Karagözoğlu et al. 2024 (Turkey) [24]	PBM laser, next-generation diode device (GRR) + pharmacological therapy (*n* = 15) PBM laser, Nd: YAG device + pharmacological therapy (*n* = 15) Sham laser + pharmacological therapy (*n* = 15)	**PBM laser GRR** Diode laser with rectangular probe, outer surface of 60 mm, inner surface of 30 mm, and multiple diodes, combining 22 mW at 904 nm and 10 mW and 650 nm, delivering 16 J per minute, with 5-minute exposure along the trigeminal branches. Total of 25 sessions: once daily for 5 consecutive days, followed by 10 sessions on alternate days. The treatment was then paused for one week and resumed with once-daily sessions for 5 days, followed by an additional 5 sessions on alternate days. **PBM laser Nd: YAG** Nd: YAG laser, spot size 0.9 mm in diameter, 1064 nm wavelength, 0.25 W output power, 5 J per point, 8 J/cm² along the nerve pathway, 20 s of exposure per point, applied to 3 points, totaling 60 s of exposure (20 s per point), 3 sessions per week. Pharmacological Therapy* Carbamazepine	**PBM laser GRR** About de 7 weeks (10 sessions) **PBM Nd: YAG and sham laser** 4 weeks (12 sessions) Follow-up: NI	A significant difference was observed between pre- and post-treatment values across all analyzed outcomes (*P* < 0.05). Treatment with the GRR laser was more effective than both the Nd: YAG laser and the placebo in reducing pain frequency, pain intensity, and interference with specific facial activities (*P* < 0.001). No significant difference was found between the groups regarding interference with general activities.

*Dosage not described in the study. *PBM* photobiomodulation, *M* male, *F* female, *NANTCL* non-ablative, non-thermal CO₂ laser, *VAS* Visual Analog Scale, *CMAP* compound muscle action potential

Sham laser was employed as a control intervention in the studies by Walker et al. [26], Waked et al. [21], Amanat et al. [19], Ebrahimi et al. [22], Rezazadeh et al. [23], and Karagözoğlu et al. [24]. Antonić et al. [20] compared different laser PBM protocols. Although sham laser was used as a control, both Waked et al. [21] and Karagözoğlu et al. [24] also investigated variations in PBM protocols or laser devices. Al-Azab et al. [25] assigned participants to three treatment groups: one receiving pharmacological therapy alone, another receiving electromagnetic therapy in combination with pharmacological treatment, and a third group treated with PBM combined with pharmacological agents.

In most of the included studies, laser PBM was administered using infrared wavelengths [19, 22, 25, 27]. Karagözoğlu et al. [24] used a laser device that provides infrared and red wavelengths at same time. Antonić et al. [20] compared red (660 nm) and infrared (830 nm) wavelengths, reporting greater symptom reduction with the longer wavelength. PBM parameters are detailed in Table 2, which shows that energy densities ranged from 3 J/cm² [20] to 12.73 J/cm² [19], and total energy per point ranged from 3 J to 16 J. Six studies used low-power devices [19, 20, 22, 24–26], and one study employed a defocused high-power laser device [23]. In two studies, the output power was not reported [21, 27]. The number of treatment sessions varied from six [24] to thirty [26], typically administered on alternating days, with treatment frequencies ranging from three [19, 21–23, 25–27] to five sessions per week [20]. Karagözoğlu et al. [24] adopted a distinct treatment frequency protocol, further detailed in Table 2. Follow-up assessments were not reported in four studies [20, 21, 24–26]. Additionally, PBM parameters were incompletely or insufficiently described in the studies by Aghamohammadi et al. [27], Waked et al. [21], and Al-Azab et al. [25].

All studies, with the exception of Amanat et al. [19], reported that the reduction in pain scores was significantly greater in the laser-treated groups compared to the control groups. Rezazadeh et al. [23] observed that the difference in pain score reduction between the laser and sham laser groups was statistically significant only up to one week after the end of treatment. Conversely, Aghamohammadi et al. [27] associated the use of PBM with an extended symptom-free period following intervention. Notably, even among groups receiving sham laser, a significant reduction in pain symptoms was observed.

## Risk of bias

**Fig. 2:**
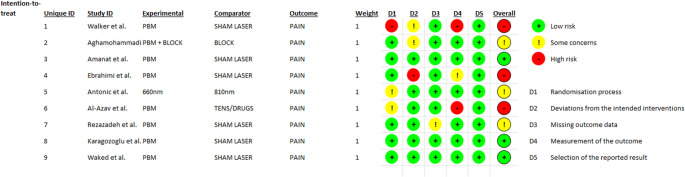
Risk of bias summary: review authors’ judgements about each risk of bias item for each included study

**Fig. 3:**
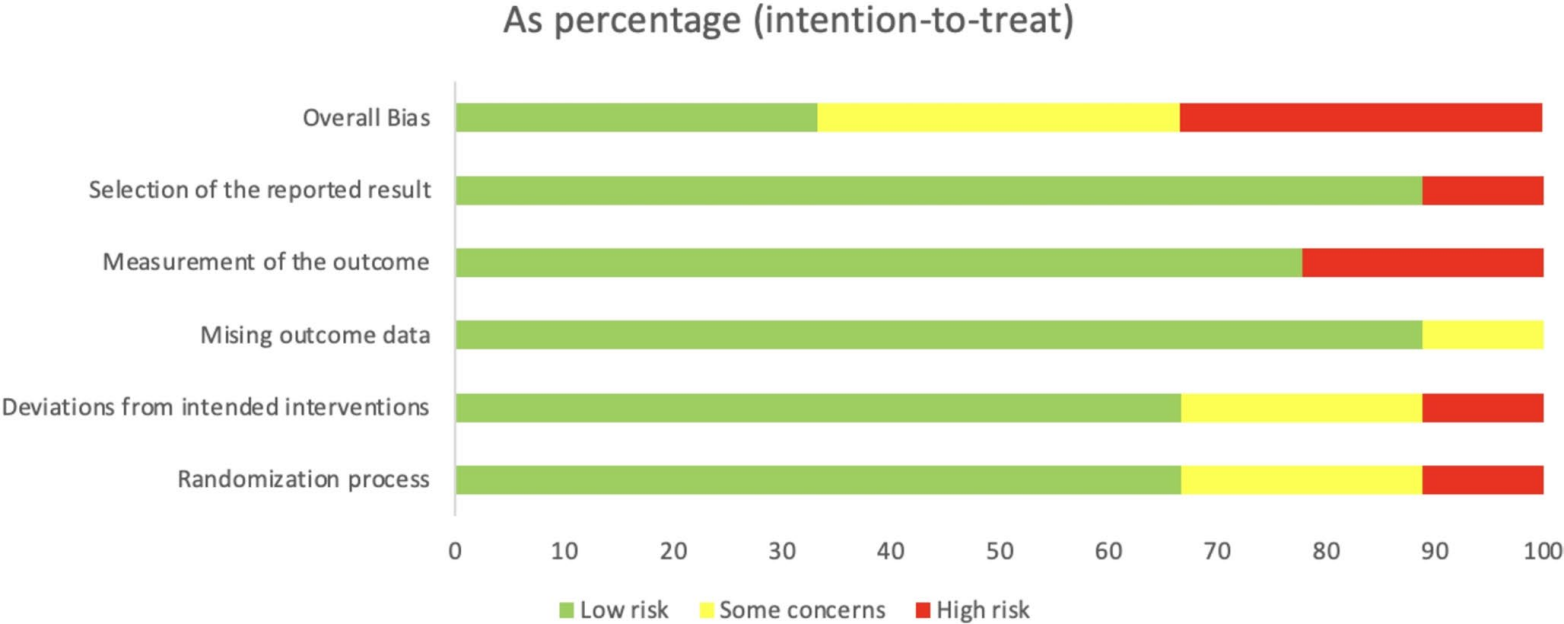
Risk of bias graph: review authors’ judgements about each risk of bias item presented as percentages across all included studies

Figures 2 and 3 provide a detailed summary of the risk of bias assessment, performed using the Cochrane Risk of Bias 2.0 (RoB 2.0) tool, which encompasses five methodological domains applicable to randomized controlled trials. The evaluation included nine studies and was conducted in accordance with the intention-to-treat principle.

In the domain of *Randomization Process*, 66.7% of the studies were classified as having low risk of bias, 22.2% raised some concerns, and 11.1% were considered at high risk. This indicates that, although most studies employed appropriate allocation methods, a portion lacked clarity or sufficient control over this process. Regarding *Deviations from Intended Interventions*, the same distribution was observed, suggesting that most studies adhered adequately to the planned intervention protocols. In the domain of *Missing Outcome Data*, the results were more favorable: 88.9% of the studies were classified as low risk of bias, while only 11.1% raised some concerns. This suggests a low rate of loss to follow-up or incomplete data across the included studies. Regarding *Measurement of Outcomes*, 77.8% of the studies were classified as having low risk of bias, while 22.2% were assessed as having high risk. Although most studies employed validated and consistent instruments, some exhibited methodological shortcomings related to the objectivity or blinding of pain assessment. In the domain of *Selection of Reported Results*, 88.9% of the studies were rated as low risk, and 11.1% as high risk. This indicates that, in the majority of cases, the outcomes that had been pre-specified were appropriately reported.

Finally, regarding the overall risk of bias, a balanced distribution was observed: one-third of the studies were classified as high risk, and one-third as low risk. Although a portion of the evidence demonstrated methodological rigor, the remaining studies presented limitations that should be considered when interpreting the results of the meta-analysis.

### Quantitative analysis

**Fig. 4:**
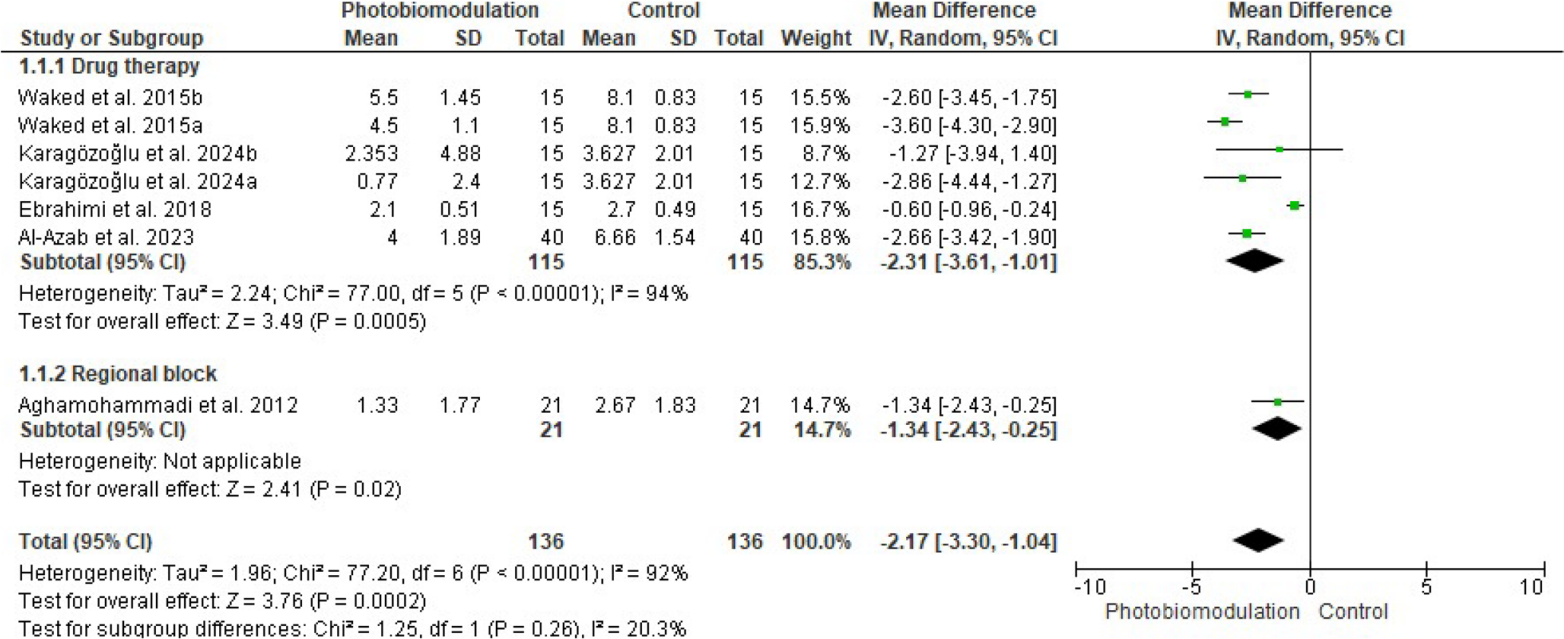
Forest plot of the effects of photobiomodulation (PBM) on pain scores in patients with trigeminal neuralgia

Of the nine studies investigating the effect of PBM in the management of trigeminal neuralgia, five were included in the meta-analysis (Fig. 4). To minimize potential confounding factors, the initial analysis included four studies [21, 22, 24, 25] in which all participants were treated with carbamazepine. Sham laser [21, 22, 24] or no treatment [25] was used as the control condition besides the pharmacological treatment. PBM combined with carbamazepine significantly reduced pain compared to pharmacotherapy alone (MD − 2.31; 95% CI, − 3.61 to − 1.01; *P* = 0.0005). PBM combined with carbamazepine resulted in an approximate 53% reduction in pain intensity, compared with the 21% reduction observed with the medication alone. However, substantial heterogeneity was detected among the studies (I² = 94%), suggesting considerable variability.

When the meta-analysis was expanded (Fig. 4) to include one additional study in which regional blockade of the Gasserian ganglion was employed [27], the results remained significant in favor of PBM for pain reduction (MD − 2.17; 95% CI, − 3.30 to − 1.04; *P* = 0.0002). Nevertheless, heterogeneity across studies remained high (I² = 92%).

**Fig. 5:**
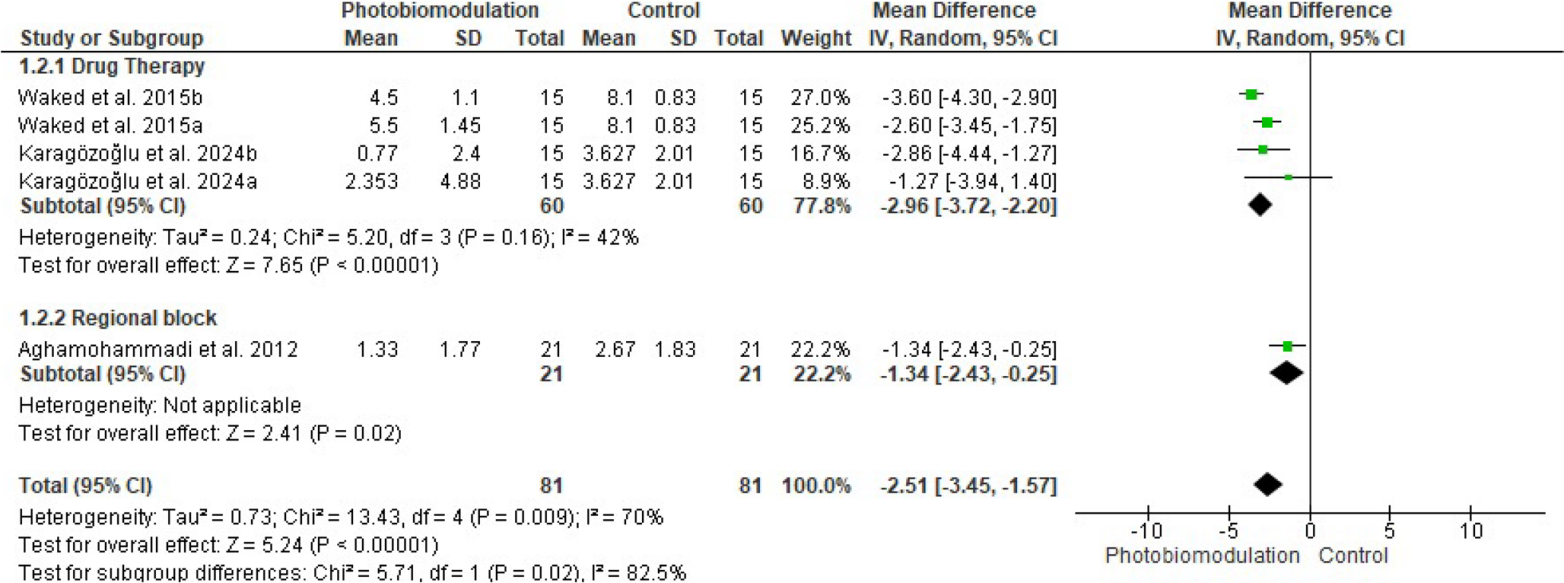
Forest plot of the effects of photobiomodulation (PBM) on pain scores in patients with trigeminal neuralgia, excluding studies with a high risk of bias

Sensitivity analysis revealed that heterogeneity among studies decreased when excluding those with a high risk of bias [22, 25] from the meta-analysis. In Fig. 5, the initial analysis included two studies [21, 24], demonstrating that PBM combined with carbamazepine significantly reduced pain compared to pharmacotherapy alone (MD − 2.96; 95% CI, − 3.72 to − 2.20; *P* < 0.00001), with moderate heterogeneity observed (I² = 42%). When the study by Aghamohammadi et al. [27] was added, the significant effect in favor of PBM persisted (MD − 2.51; 95% CI, − 3.45 to − 1.57; *P* < 0.00001), but heterogeneity increased (I² = 70%).

## Discussion

This systematic review evaluated the efficacy of laser PBM, whether used as a standalone therapy or as an adjuvant to other treatments, in reducing pain in patients with trigeminal neuralgia. Within the context of chronic orofacial pain, this condition is characterized by its disabling nature and presents significant diagnostic and therapeutic challenges, particularly when it becomes refractory to conventional treatments [28]. This review also aimed to assess the efficacy of PBM in the management of postherpetic neuralgia. Although numerous studies have investigated this therapy, none have met the inclusion criteria. In most of these studies, participants with postherpetic neuralgia affecting various anatomical regions were included, making it impossible to isolate and analyze data specific to the orofacial region.

A sample of 387 patients with trigeminal neuralgia was included in the qualitative analysis. PBM, whether applied as a monotherapy [20, 26], in combination with centrally acting pharmacological agents [19, 21–25], or alongside ganglion block [27], and administered either along the nerve pathway or at trigger points, demonstrated efficacy in reducing pain scores. Moreover, through quantitative analysis, this review confirmed that laser PBM significantly reduced pain in patients with trigeminal neuralgia. The magnitude of improvement achieved with the addition of PBM to conventional treatment (carbamazepine) reinforces the clinical relevance of the findings of this study. Laser PBM triggers cellular responses that include reduction of inflammation, modulation of neural excitability thresholds, and enhancement of local microcirculation, all of which contribute to pain relief [29]. In addition to modulating the inflammatory response, PBM promotes tissue regeneration, supporting the recovery of neuromuscular structures that are frequently affected. PBM also exerts an analgesic effect in inflamed peripheral tissues, suggesting the involvement of the endogenous opioid system. Further evidence indicates that this therapy increases the expression of messenger RNA precursors of endorphins in both in vitro and in vivo models [27]. There is also evidence that laser PBM selectively inhibits action potentials in small sensory fibers when applied at precise dosages, effectively blocking nociceptors to “switch off” pain while preserving the function of large sensory fibers (maintaining non-painful touch) and motor fibers [30, 31]. Jenkins et al. [30] proposed that this selective blockade of electrical activity in small-diameter fibers is significant and warrants its own subcategory, termed *Transient Selective Neural Inhibition via PBM*. These effects are reversible and occur without adverse effects or damage to neural tissue.

Haghighat et al. [32] also conducted a systematic review to assess the efficacy of PBM in the treatment of trigeminal neuralgia. However, the included studies encompassed participants with heterogeneous orofacial pain conditions, which precluded the identification of the exact number of individuals affected by trigeminal neuralgia. Moreover, several studies included in that review involved neural ablation using CO₂ laser rather than PBM. These limitations compromise the accuracy and specificity of the findings reported. In another systematic review conducted by Ibarra et al. [33], 193 patients with trigeminal neuralgia were included. Despite the positive effects of PBM on trigeminal neuralgia, no meta-analysis could be conducted. More recently, Díaz et al. [34] carried out a systematic review to evaluate the effectiveness of laser PBM in the management of maxillofacial neuropathies, including neuralgias, paralysis, and paresthesia. A detailed description of the included studies or the meta-analysis was not provided, despite positive results being reported. Therefore, the present review is pioneering in presenting quantitative data on the efficacy of PBM in managing pain in patients with trigeminal neuralgia.

Due to the severity of pain symptoms, participants with trigeminal neuralgia in seven of the nine included studies were receiving centrally acting pharmacological agents, such as carbamazepine or gabapentin. These medications are associated with a range of adverse effects, including drowsiness, nausea, vomiting, headache, and xerostomia, in addition to more serious risks such as blood dyscrasias and erythema multiforme, among others [16]. Aghamohammadi et al. [27] demonstrated that PBM allowed for a significant reduction in the daily dosage of carbamazepine. All studies included in this review unanimously reported the absence of adverse effects associated with laser PBM. Given the potential complications related to pharmacological therapies, these findings suggest that PBM may also contribute to reducing the dosage and complexity of drug regimens required for the management of trigeminal neuralgia.

All included studies assessed patient-reported pain levels before and after treatment. However, since five of these studies did not report follow-up data [20, 21, 24–26], only pain scores at the end of treatment were used for comparative purposes. Despite the overall positive findings regarding the efficacy of PBM in managing trigeminal neuralgia, studies by Amanat et al. [19], Ebrahimi et al. [22], and Rezazadeh et al. [23] reported a tendency toward pain recurrence following the completion of therapy. This observation aligns with the chronic nature of the condition and suggests that additional PBM treatment cycles may be necessary depending on individual patient needs. Other outcomes assessed in the included studies included the amplitude of compound muscle action potentials (CMAPs) [25] and the degree of interference in general and specific facial activities [24]. Al-Azab et al. [25] observed that PBM led to improvements in the amplitude of masseter and temporalis CMAPs. Conversely, Karagözoğlu et al. [24] found no significant effects of PBM on orofacial functional outcomes. These findings highlight the need for further research investigating the effects of laser PBM on broader clinical endpoints such as muscle function and quality of life.

Another noteworthy finding is that even in groups treated with sham laser, a significant reduction in pain was observed at the end of treatment. The placebo effect associated with the use of simulated laser—commonly referred to as sham laser—may be attributed to psychological factors such as the expectation of relief, the clinical setting, and prior conditioning of the patient. Despite the absence of active radiation emission, sham laser may activate endogenous pain modulation pathways, including the opioid system, thereby reducing pain perception. Elements such as the technological appearance of the equipment and the professional-patient relationship also reinforce the placebo response [35].

The risk of bias assessment revealed heterogeneous methodological quality among the included studies, which compromises the overall strength of the available evidence. Although one-third of the studies were classified as having low risk of bias [19, 21, 24], a substantial proportion were assessed as having high risk [22, 25, 26] or raising methodological concerns [20, 23, 27]. These findings indicate weaknesses in randomization procedures, outcome blinding, and overall protocol execution. These results underscore the need for future randomized controlled trials that are better designed to establish more robust evidence regarding the clinical use of laser PBM in the management of trigeminal neuralgia.

The present systematic review identified significant methodological limitations within the included studies, which directly affect the generalizability of the findings. Several clinical trials lacked complete data such as means, standard deviations, and p-values, hindering the inclusion of these studies in more precise quantitative analyses [19, 20, 23, 26]. Moreover, a recurring issue was the insufficient reporting of detailed PBM parameters—information that is essential for both clinical and scientific reproducibility. To address the variability in PBM protocols and the lack of standardized reporting, Esteves-Pereira et al. [36] advocate for the systematic inclusion of core parameters in all clinical and experimental studies. These parameters include: wavelength, output power, irradiation time (in seconds) per point, radiant energy, fluence, irradiance, mode of operation, application technique (static versus optical scanning), anatomical location, number of sessions, and treatment interval. Moreover, factors such as the optical spot size at the tissue surface, beam divergence angle, distance from the target, and the use of a power meter to calibrate the optical source are of utmost importance when considering laser PBM therapy [37]. In an effort to standardize PBM protocols globally, the World Association for Photobiomodulation Therapy (WALT) proposed the use of the Einstein (E) unit [38]. More recently, Esteves-Pereira et al. [36] recommended the adoption of this unit to enhance the clarity and uniformity of PBM prescription and communication. By integrating concepts such as irradiance, fluence, radiant energy, photon energy, and photonic fluence, the Einstein unit transcends geographical and linguistic boundaries, enabling professionals worldwide to operate within a unified framework. The adoption of this standardization represents a scientific advance in the field of laser therapy.

Another limitation identified in this review was the small sample size observed in many of the included studies, which compromises statistical power and increases the risk of publication bias. Additionally, the natural variability of trigeminal neuralgia, which may include periods of spontaneous remission, may influence the reported pain outcomes and should be taken into account when interpreting the effectiveness of PBM. Furthermore, the randomized clinical trials included in this review did not provide information to distinguish trigeminal neuralgia caused by vascular compression from idiopathic cases, preventing analyses based on etiological subtypes. Together, these methodological shortcomings underscore the need for future research with more rigorous study designs, greater standardization, and enhanced transparency in the reporting of therapeutic protocols.

## Conclusion

Despite the heterogeneity among the studies, the findings suggest that PBM is effective as an adjuvant therapy in the management of trigeminal neuralgia, demonstrating a favorable safety profile and potential to reduce medication dependence. However, the lack of standardization in treatment protocols, small sample sizes, and absence of long-term follow-up are recurring limitations that should be addressed in future research.

## Supplementary Information

Below is the link to the electronic supplementary material.

Supplementary Material 1 (DOCX 17.7 KB) 

## Data Availability

The data that support the findings of this study are available from the corresponding author, [FGS], upon reasonable request.

## Declarations

### Study Registration

This study was previously registered on the International Prospective Register of Systematic Reviews under registration number CRD420251102473.
