# Supplementary material for: Effectiveness of laser photobiomodulation in the management of trigeminal neuralgia: a systematic review and meta-analysis
Source: Lasers Med Sci. 2026 Feb 3;41(1):19. doi: 10.1007/s10103-026-04804-9 (PMC12864350; doi:10.1007/s10103-026-04804-9)
Supplement: Supplementary file 1 — Supplementary Material 1 (DOCX 17.7 KB) [file 10103_2026_4804_MOESM1_ESM.docx]

**Specific search terms for each database:**

| **DATA BASE** | **SEARCH TERMS** |
| --- | --- |
| PUBMED | “Facial Neuralgia” OR “Facial Pain” OR “Trigeminal Neuralgia” OR “Neuralgia, Postherpetic” AND “Laser therapy” OR “Low Level Light Therapy” |
| EMBASE | 'Face Pain' OR 'Trigeminus Neuralgia' OR 'Postherpetic Neuralgia' OR 'Myofascial Pain' AND 'Low Level Laser Therapy' |
| WEB OF SCIENCE | “Facial Neuralgia” OR “Facial Pain” OR “Trigeminal Neuralgia” OR “Neuralgia, Postherpetic” AND “Laser therapy” OR “Low Level Light Therapy” |
| LILACS | “Facial Neuralgia” OR “Facial Pain” OR “Trigeminal Neuralgia” OR “Neuralgia, Postherpetic” AND “Laser therapy” OR “Low Level Light Therapy” |
| SCOPUS | “Facial Neuralgia” OR “Facial Pain” OR “Trigeminal Neuralgia” OR “Neuralgia, Postherpetic” AND “Laser therapy” OR “Low Level Light Therapy” |
| COCHRANE LIBRARY | “Facial Neuralgia” OR “Facial Pain” OR “Trigeminal Neuralgia” OR “Neuralgia, Postherpetic” AND “Laser therapy” OR “Low Level Light Therapy” |

**SEARCH STRATEGY - PUBMED**

**#1:**

“Facial Neuralgia”[MeSH] OR “Facial Neuralgia” OR “Facial Neuralgias” OR “Neuralgia, Facial” OR “Neuralgias, Facial” OR “Facial Pain Syndromes” OR “Facial Pain Syndrome” OR “Pain Syndrome, Facial” OR “Pain Syndromes, Facial” OR “Syndrome, Facial Pain” OR “Syndromes, Facial Pain” OR “Craniofacial Pain Syndromes” OR “Craniofacial Pain Syndrome” OR “Pain Syndrome, Craniofacial” OR “Pain Syndromes, Craniofacial” OR “Syndrome, Craniofacial Pain” OR “Syndromes, Craniofacial Pain” OR “Sphenopalatine Neuralgia” OR “Neuralgia, Sphenopalatine” OR “Neuralgias, Sphenopalatine” OR “Sphenopalatine Neuralgias” OR "Myofascial Pain Syndromes"[Mesh] OR "Myofascial Pain Syndromes" OR “Myofascial Pain Syndrome” OR “Pain Syndrome, Myofascial” OR “Pain Syndromes, Myofascial” OR “Syndrome, Myofascial Pain” OR “Syndromes, Myofascial Pain” OR “Myofascial Trigger Point Pain” OR “Trigger Point Pain, Myofascial” OR “Myofascial Pain Dysfunction Syndrome” OR “Facial Pain”[MeSH] OR “Facial Pain” OR “Pain, Facial” OR “Face Pain” OR “Pain, Face” OR “Craniofacial Pain” OR “Pain, Craniofacial” OR “Myofacial Pain” OR “Pain, Myofacial” OR “Orofacial Pain” OR “Pain, Orofacial” OR “Neuralgic Facial Pain” OR “Facial Pain, Neuralgic” OR “Pain, Neuralgic Facial” OR “Trigeminal Neuralgia”[MeSH] OR “Trigeminal Neuralgia” OR “Neuralgia, Trigeminal” OR “Trigeminal Neuralgias” OR “Tic Douloureux” OR “Epileptiform Neuralgia” OR “Epileptiform Neuralgias” OR “Neuralgia, Epileptiform” OR “Fothergill Disease” OR “Disease, Fothergill” OR “Trifacial Neuralgia” OR “Neuralgia, Trifacial” OR “Trifacial Neuralgias” OR “Tic Doloureux” OR “Secondary Trigeminal Neuralgia” OR “Neuralgia, Secondary Trigeminal” OR “Secondary Trigeminal Neuralgias” OR “Trigeminal Neuralgia, Secondary” OR “Trigeminal Neuralgia, Idiopathic” OR “Idiopathic Trigeminal Neuralgia” OR “Idiopathic Trigeminal Neuralgias” OR “Neuralgia, Idiopathic Trigeminal” OR “Neuralgia, Postherpetic”[MeSH] OR “Neuralgia, Postherpetic” OR “Postherpetic Neuralgia”

**#2:**

“Laser therapy”[MeSH] OR “Laser therapy” OR “Laser Therapies” OR “Therapies, Laser” OR “Therapy, Laser” OR “Nonablative Laser Treatment” OR “Laser Treatment, Nonablative” OR “Laser Treatments, Nonablative” OR “Nonablative Laser Treatments” OR “Low Level Light Therapy”[MeSH] OR “Low Level Light Therapy” OR “Light Therapies, Low-Level” OR “Light Therapy, Low-Level” OR “Low-Level Light Therapies” OR “Low Level Light Therapy” OR “Therapies, Low-Level Light” OR “Therapy, Low-Level Light” OR “LLLT” OR “Photobiomodulation Therapy” OR “Photobiomodulation Therapies” OR “Therapies, Photobiomodulation” OR “Therapy, Photobiomodulation” OR “Photobiomodulation” OR “Photobiomodulations” OR “Laser Therapy, Low-Level” OR “Laser Therapies, Low-Level” OR “Laser Therapy, Low Level” OR “Low-Level Laser Therapies” OR “Laser Biostimulation” OR “Biostimulation, Laser” OR “Laser Irradiation, Low-Power” OR “Irradiation, Low-Power Laser” OR “Laser Irradiation, Low Power” OR “Laser Phototherapy” OR “Phototherapy, Laser” OR “Laser Therapy, Low-Power” OR “Laser Therapies, Low-Power” OR “Laser Therapy, Low Power” OR “Low-Power Laser Therapies” OR “Low-Level Laser Therapy” OR “Low Level Laser Therapy” OR “Low-Power Laser Irradiation” OR “Low Power Laser Irradiation” OR “Low-Power Laser Therapy” OR “Low Power Laser Therapy”

**#3**

#1 AND #2

**SEARCH STRATEGY -EMBASE**

**#1**

('face pain'/exp OR 'face neuralgia' OR 'face pain' OR 'facial neuralgia' OR 'facial pain' OR 'facialgia' OR 'neuralgia, face' OR 'paroxysmal facial pain' OR 'trigeminus neuralgia'/exp OR 'fifth cranial nerve pain' OR 'mandibular nerve pain' OR 'mandibular neuropathic pain' OR 'neuralgia, trigeminus' OR 'prosopalgia' OR 'prosoponeuralgia' OR 'tic douloureux' OR 'trigeminal nerve neuralgia' OR 'trigeminal nerve pain' OR 'trigeminal neuralgia' OR 'trigeminal neuropathic pain' OR 'trigeminus nerve neuralgia' OR 'trigeminus neuralgia' OR 'postherpetic neuralgia'/exp OR 'herpetic neuralgia' OR 'neuralgia, postherpetic' OR 'postherpetic neuralgia' OR 'postherpetic pain' OR 'myofascial pain'/exp OR 'myofascial pain' OR 'myofascial pain dysfunction' OR 'myofascial pain dysfunction syndrome' OR 'myofascial pain syndrome' OR 'myofascial pain syndromes' OR 'myofascial syndrome')

**#2**

('low level laser therapy'/exp OR 'lilt (laser therapy)' OR 'lllt (laser therapy)' OR 'low-intensity (therapeutic) laser therapy (lilt)' OR 'cold laser therapy' OR 'laser biostimulation' OR 'laser therapy, low-level' OR 'low energy laser therapy' OR 'low energy laser treatment' OR 'low intensity laser therapy' OR 'low intensity laser treatment' OR 'low level laser therapy' OR 'low level laser treatment' OR 'low level light therapy' OR 'low power laser therapy' OR 'low power laser treatment' OR 'low-level laser therapy' OR 'low-level laser therapy (lllt)' OR 'low-level light therapy' OR 'photo biomodulation therapy' OR 'photo-bio-modulation therapy' OR 'photo-biomodulation (pbm) therapy' OR 'photo-biomodulation therapy (pbmt)' OR 'photobiomodulation (pbm) therapy' OR 'photobiomodulation therapy' OR 'photobiomodulation therapy (pbm)' OR 'photobiomodulation therapy (pbmt)' OR 'soft laser therapy' OR 'therapeutic laser therapy')

**#3**

#1 AND #2

**SEARCH STRATEGY - Web of Science/Scopus/Cochrane Library/LILACS**

**#1**

“Facial Neuralgia” OR “Neuralgia, Facial” OR “Facial Pain Syndrome” OR “Craniofacial Pain Syndrome” OR “Sphenopalatine Neuralgia” OR “Facial Pain” OR “Craniofacial Pain” OR “Neuralgic Facial Pain” OR “Trigeminal Neuralgia” OR “Neuralgia, Trigeminal” OR “Tic Douloureux” OR “Epileptiform Neuralgia” OR “Fothergill Disease” OR “Trifacial Neuralgia” OR “Secondary Trigeminal Neuralgia” OR “Trigeminal Neuralgia, Idiopathic” OR “Neuralgia, Postherpetic” OR “Postherpetic Neuralgia”

**#2**

“Laser therapy” OR “Laser Therapies” OR “Therapies, Laser” OR “Therapy, Laser” OR “Nonablative Laser Treatment” OR “Low Level Light Therapy” OR “Light Therapies, Low-Level” OR “Light Therapy, Low-Level” OR “Low-Level Light Therapies” OR “Low Level Light Therapy” OR “Therapies, Low-Level Light” OR “Therapy, Low-Level Light” OR “LLLT” OR “Photobiomodulation Therapy” OR “Therapies, Photobiomodulation” OR “Therapy, Photobiomodulation” OR “Photobiomodulation” OR “Laser Therapy, Low-Level” OR “Laser Therapies, Low-Level” OR “Laser Therapy, Low Level” OR “Low-Level Laser Therapies” OR “Laser Biostimulation” OR “Laser Irradiation, Low-Power” OR “Irradiation, Low-Power Laser” OR “Laser Irradiation, Low Power” OR “Laser Phototherapy” OR “Laser Therapy, Low-Power” OR “Laser Therapies, Low-Power” OR “Laser Therapy, Low Power” OR “Low-Power Laser Therapies” OR “Low-Level Laser Therapy” OR “Low Level Laser Therapy” OR “Low-Power Laser Irradiation” OR “Low Power Laser Irradiation” OR “Low-Power Laser Therapy” OR “Low Power Laser Therapy”

**#3**

#1 AND #2
